# Assessment of Blood Loss during Neuroendovascular Procedures

**DOI:** 10.3390/jcm13030677

**Published:** 2024-01-24

**Authors:** Michael Goutnik, Andrew Nguyen, Chance Fleeting, Aashay Patel, Brandon Lucke-Wold, Dimitri Laurent, Tamara Wahbeh, Shawna Amini, Fadi Al Saiegh, Matthew Koch, Brian Hoh, Nohra Chalouhi

**Affiliations:** 1 Department of Neurosurgery, University of Florida, Gainesville, FL 32601, USA; mgoutnik@ufl.edu (M.G.); andrewnguyen@ufl.edu (A.N.); cfleeting@ufl.edu (C.F.); aashay.patel@surgery.ufl.edu (A.P.); tamara.wahbeh@neurosurgery.ufl.edu (T.W.);; 2 Department of Neurosurgery, University of Texas Health Science Center, San Antonio, TX 78229, USA

**Keywords:** neuroendovascular procedures, anemia, hemoglobin, angiography

## Abstract

(1) Background: Neuroendovascular procedures have generally been considered to have minor or inconsequential blood loss. No study, however, has investigated this question. The purpose of this study is to quantify the blood loss associated with neuroendovascular procedures and identify predictors of blood loss, using hemoglobin change as a surrogate for blood loss. (2) Methods: A retrospective review of 200 consecutive endovascular procedures (diagnostic and therapeutic) at our institution from January 2020 to October 2020 was performed. Patients had to have pre- and post-operative hematocrit and hemoglobin levels recorded within 48 h of the procedure (with no intervening surgeries) for inclusion. (3) Results: The mean age of our cohort was 60.1 years and the male representation was 52.5%. The mean pre-operative hemoglobin/hematocrit was significantly lower among females compared to males (12.1/36.2 vs. 13.0/38.5, *p* = 0.003, *p* = 0.009). The mean hemoglobin decrease was 0.5 g/dL for diagnostic angiograms compared to 1.2 g/dL for endovascular interventions (*p* < 0.0001), and 1.0 g/dL for all procedures combined. In a multivariate linear regression analysis, pre-operative antiplatelet/anticoagulant use was associated with a statistically significant decrease in hemoglobin. (4) Conclusions: Our data support that blood loss from diagnostic angiograms is marginal. Blood loss in endovascular interventions, however, tends to be higher. Pre-operative blood antiplatelet/anticoagulant use and increasing age appear to increase bleeding risk and may require closer patient monitoring.

## 1. Introduction

Cerebral angiography remains the gold standard for diagnosing cerebrovascular lesions. The main advantage over computed tomography angiography (CTA) or magnetic resonance angiography (MRA) is its ability to provide an enhanced visualization of the cerebral vasculature. This includes the ability to detect subtle structural abnormalities such as small, blister, and mycotic aneurysms that could be missed on CTA or MRA. Additionally, it allows for the real-time visualization of cerebral blood flow from the arterial to capillary to venous stage [1]. This allows for the assessment, diagnosis, and surgical planning for vascular abnormalities including thromboembolic occlusions, flow-decreasing stenosis, aneurysms, arteriovenous malformations (AVMs), and dural arteriovenous fistulae (dAVFs) [2]. Despite the ubiquitous use of cerebral angiography, there has not been extensive investigation into the blood loss occurring during diagnostic angiography and endovascular procedures. The premise is that bleeding is assumed to be minimal or clinically inconsequential for most patients, with a low transfusion rate [3]. With saline infusions, frequent catheter exchanges, and aspiration during thrombectomies, it can be difficult to accurately quantify blood loss. Considering the frequently overestimated and subjective visual measurements of blood loss performed in the operating room [4,5], surrogate markers such as hematocrit and hemoglobin were used in this study to quantify pre- and post-procedural changes [6]. Therefore, the purpose of this study was two-fold: (a) to characterize the change in hematocrit and hemoglobin after neuroendovascular procedures and (b) to identify the factors that are associated with excessive hemoglobin change to encourage more careful monitoring. 

## 2. Materials and Methods

Approval was obtained from the Institutional Review Board at the University of Florida (protocol code IRB202202029, 3/29/23). Data were sequentially collected (by 4 authors, M.G., A.N., C.F., and A.P.) from 200 patients undergoing diagnostic angiography or endovascular interventions, beginning January 2020 and extending to October 2020. The data were extracted from EPIC electronic health record and transferred to Research Electronic Data Capture (REDCap). A total of 19 patients were excluded. Patients were excluded if data were missing, including if there were no pre-operative or post-operative hemoglobin and hematocrit measurements documented within 48 h of surgery. Patients were also excluded if there were significant operative interventions preceding the post-angiography complete blood count (CBC). 

Data collected included patient demographics (age, race, gender, height, and weight), procedural details (including type of procedure, duration, known pre-operative antiplatelet/anticoagulant use, arterial access site, sheath size, post-operative disposition, and post-operative complications), and pre- and post-operative CBC values (including hemoglobin, hematocrit, red blood cell count, and white blood count).

Statistical analysis, including paired *t*-tests, Pearson’s chi-squared test, and multivariable regression, was conducted via JMP Pro version 14. The multivariate regression involved hemoglobin decrease as the response variable, with various demographic, procedural, and CBC variables as predictors. The CBC variables of hematocrit, white blood cell, and red blood cell counts were not included in the multivariate analysis due to correlation with hemoglobin. To avoid overfitting due to 14 separate levels of endovascular intervention and combinations of interventions, a binary variable of treatment type (diagnostic angiography/no treatment vs. intervention) was created and used in multivariate analysis. 

## 3. Results

### 3.1. Baseline Characteristics

The mean age of the cohort was 60.1 years with 105 (52.5%) males. Of the patients, 96 (48%) had been on an antiplatelet or anticoagulant medication prior to surgery. The mean pre-operative hemoglobin and hematocrit were 12.6 g/dL and 37.4%, respectively. The mean pre-operative hemoglobin and hematocrit differed significantly among females compared to males (12.1/36.2 vs. 13.0/38.5, *p* = 0.003, *p* = 0.009). While 37 (38.9%) women had pre-operative anemia (hemoglobin below 12 g/dL), 28 (26.7%) men had pre-operative anemia (hemoglobin below 12.5 g/dL). Of the patients, 62 (31.0%) had diagnostic angiograms without intervention, while 138 (69.0%) had endovascular interventions including coil embolization, onyx embolization, flow diversion, or thrombectomy. The overall mean surgical duration was 80.5 min. The mean duration for diagnostic angiography was 56.5 min while the mean duration for endovascular interventions was 91.3 min (*p* < 0.0001). The baseline characteristics are summarized in Table 1. 

**Figure 1:**
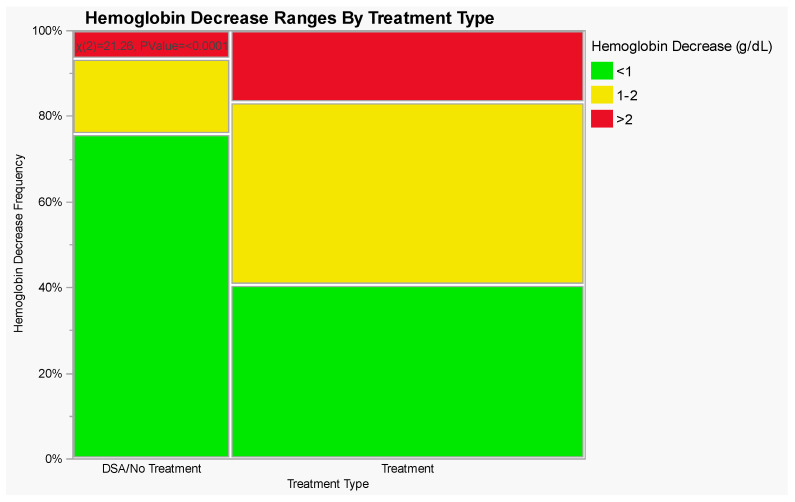
Ranges of hemoglobin decrease by treatment type. Visual representation of Table 2.

The mean hemoglobin decrease was 1.0 g/dL, while the mean hematocrit decrease was 2.8% for all procedures combined. The mean hemoglobin decrease was 0.5 g/dL for diagnostic angiograms compared to 1.2 g/dL for endovascular interventions (*p* < 0.0001). Only 4 (6.5%) patients in the diagnostic group versus 23 (16.6%) in the interventional group had a hemoglobin decrease of greater than 2 g/dL (*p* = 0.0506). Only 2 (1.0%) of 200 patients had a post-operative hemoglobin of <7 g/dL; their pre-operative hemoglobins were also low (<7.6 g/dL) and one of these patients was on an antiplatelet/anticoagulant medication pre-operatively. A summary of hemoglobin differences between diagnostic angiograms and endovascular interventions is provided in Table 2 and Figure 1.

Five (2.5%) patients had post-operative complications. Of these, three had intracranial hemorrhage, one had groin hematoma, and one ultimately expired during hospitalization. The mean hemoglobin/hematocrit decrease between the no complication and complication groups were 0.9/2.7 and 1.8/5.6, respectively (*p* = 0.057). 

Patients taking pre-operative anticoagulants/antiplatelet medications had a mean hemoglobin decrease of 1.2 g/dL, while patients not taking one of those medications had a mean hemoglobin decrease of 0.7 g/dL (*p* = 0.0002). Patients younger than 60 had a mean hemoglobin decrease of 0.7 g/dL while those older than 60 had a mean decrease of 1.1 g/dL (*p* = 0.006). 

### 3.2. Regression

A multiple regression model was constructed to investigate the factors associated with hemoglobin changes. In this model, pre-operative antiplatelet/anticoagulant use (*p* = 0.007) was significantly associated with hemoglobin loss. Age was a borderline significant predictor (*p* = 0.05). Other factors including length of surgery (*p* = 0.08), gender (*p* = 0.10), treatment type (*p* = 0.29), post-operative complications (*p* = 0.40), and arterial access site (*p* = 0.71) were not found to be significant predictors. The full regression coefficient output is summarized in Table 3, with effect tests provided in Appendix A Table A1.

## 4. Discussion

Presently, extensive exploration regarding intraoperative blood loss and transfusion has been conducted in other types of surgery. In fact, post-operative anemia is frequently encountered in major surgeries [7]. The potential causes are likely multifactorial but include hemodilution, intraoperative blood loss, and decreased erythropoiesis [8]. It has been shown that peri-operative and post-operative anemia can have potentially serious adverse effects on patients, including increasing post-operative hospital stay, increasing the risk of post-operative infections and transfusion reactions, and decreasing overall survival [7,9]. Post-operative anemia has also been linked to pulmonary, septic, thromboembolic, and wound complications [10]. A potential mechanism for post-operative anemia includes iatrogenic hemodilution from excessive peri-operative fluid administration, with hypertensive patients being potentially more vulnerable [5]. This is an important consideration as cerebrovascular patients are often hypertensive and may thus have an increased predisposition for dilutional anemia. 

While the authors’ experience and the literature suggest a relatively low bleeding requiring transfusion rate from cerebral angiography, an investigation into risk factors for bleeding was merited, considering the many inpatients and patients requiring multiple procedures that the authors treat [3]. There are no studies in the literature that have attempted to quantify blood loss in neuroendovascular procedures. A study by Rai and colleagues assessed preoperative hemoglobin and hematocrit levels only, in patients receiving outpatient cerebral angiography [11]. In their cohort, low hemoglobin, hematocrit, and platelet levels were observed in only 3.5%, 1.8%, and 4.5% of patients, respectively [11]. Our sample featured a slightly higher level of pre-operative anemia with a mean pre-operative hemoglobin level of 12.6 g/dL, with 39% of women having Hgb < 12.0 g/dL and 27% of men having <12.5 g/dL. This is likely attributable to the inclusion of many inpatients in our study, who are often more decompensated upon arrival. 

The overall results of our study suggest that diagnostic angiography and even interventional procedures are associated with generally mild blood loss, as evaluated by hemoglobin change. The mean hemoglobin loss for our cohort was only 1.0 g/dL. Studies in cardiac patients have similarly suggested blood loss following coronary angiography [12,13]. One study involving 506 patients undergoing coronary angiography and subsequent coronary artery bypass graft surgery (CABG) found that coronary angiography led to a mean hemoglobin reduction of 1.8 g/dL, predicting a need for transfusion during subsequent CABG [13]. On average, such a minor hemoglobin drop should be physiologically restored within a few weeks for most patients, with no need for transfusion [14,15,16]. For example, a study involving patients undergoing arthroplasty showed that the majority of the pre-operative hemoglobin level is restored between post-operative days 7 and 28, with a significant increase in reticulocytes by post-operative day 7 [15]. Similarly, in a study of anemic patients with acute gastrointestinal bleeding, patients with hemoglobin values between 8 and 10 g/dL at discharge had a mean 1.7 g/dL recovery at 7 days post-discharge. As such, blood loss from cerebral angiography is generally low and should be restored relatively quickly after the procedure. 

The only significant contributor to hemoglobin decrease in our cohort was pre-operative antiplatelet/anticoagulant medication usage, with age being a borderline significant variable. This parallels findings in cardiac surgery, where age and pre-operative aspirin and heparin use are variables associated with increased risk of reexploration sternotomy secondary to bleeding [17]. However, in a large meta-analysis of patients undergoing cardiovascular endovascular interventions, uninterrupted warfarin was not associated with higher odds of major bleeding compared to interrupted warfarin [18]. Similarly, a prospective analysis involving 49 patients demonstrated that those on uninterrupted direct oral anticoagulants (DOACs) during elective transradial coronary angiography did not have a difference in bleeding (overall 0% major and minor hemorrhage risks in both groups) compared to those not on DOACs [19]. This is worth further clarifying, as almost half of our sample was on a pre-operative anticoagulant/antiplatelet agent, and rates of oral anticoagulant use have been increasing among Medicare beneficiaries [20]. Other variables, such as length of surgery, sheath size, and access site, were not associated with hemoglobin change. Access site being insignificant in multivariate analysis was a surprising finding, as both coronary and cerebral angiography studies have shown increased access site complications and bleeding from femoral access compared to radial [21,22]. This may be due to our smaller sample size with an overall small number of complications. Regardless, based on the results of our study, patients on antiplatelets/anticoagulants undergoing endovascular interventions have the largest decrease in their hemoglobin post-procedurally and this should be taken into account, especially in the setting of other comorbidities. 

Our study suggests that hemoglobin change in patients undergoing interventions was more than double that of patients undergoing diagnostic angiograms. Larger sheath sizes and catheters, more exchanges, and increasing procedure time potentially mediate this difference. Furthermore, patients undergoing interventions may be on dual anti-platelet therapy (DAPT) more frequently than those receiving diagnostic angiograms [23]. Finally, it is important to consider that patients may have several endovascular interventions/diagnostic angiograms in the same hospital encounter. Thus, while the mean hemoglobin loss per procedure was mild, the cumulative loss from multiple procedures may be more significant, and is something worth investigating in future studies.

Regarding limitations, this study was retrospective, the data were gathered from a single institution, and the population was homogenous, with a high percentage of Caucasian patients in our cohort. Secondly, although all pre- and post-operative labs were collected within 48 h, there was some heterogeneity regarding the exact timing of collected labs. Ideally, the post-operative CBC would have been collected immediately following surgery. Also, the volume of saline infusion per procedure was not quantified and there may be a component of dilutional anemia, and it would thus be worth quantifying the amount of saline received during interventions as a future variable. Length of surgery was a barely insignificant variable on multivariate analysis, and may represent a surrogate for volume of infusion. In addition, surgery is known to modify circulating blood volume, so perhaps an estimate of blood loss that is not affected by volume status, such as hemoglobin mass loss (using blood from surgical sponges and suctions), may be a more accurate measurement to obtain in the future [6]. Hemoglobin mass loss does not appear to have been investigated yet in cerebral or coronary angiography. Another limitation is decreased external validity, as patients who underwent craniotomy following their endovascular procedure were excluded due to their likely influence on the post-operative CBC. In addition, an avenue of future investigation may include evaluating the effect of multiple neuroendovascular procedures, particularly in an inpatient sample. Despite these limitations, the study provides the first assessment of hemoglobin change during neuroendovascular procedures and useful guidance for clinical decision making.

## 5. Conclusions

In summary, the quantification of postoperative hemoglobin decline following routine diagnostic angiography and neuroendovascular treatments has not been carried out before. Overall, our data suggest that hemoglobin decline from routine cerebral angiography and interventional procedures is generally small, although it is significantly higher with interventions. We did find that antiplatelet/anticoagulant use and age were also likely contributing factors for decreases in hemoglobin. A careful pre-operative assessment of complication risk should therefore be performed in select cases.

## Figures and Tables

**Table 1:** Patient characteristics.

Variable	Value Categorical Variables: N (%) Continuous Variables: Mean ± SD
Age (years)	60.1 ± 15.2
Male Gender	105 (52.5)
Ethnicity	
Not Hispanic	190 (95)
Hispanic	6 (3.0)
Refused/Unknown	4 (2.0)
Race	
Asian	1 (0.5)
Black	24 (12.0)
Hispanic	3 (1.5)
Other/Unknown	8 (4.0)
White	164 (82.0)
BMI	27.8 ± 6.6
Pre-Operative Anticoagulant or Antiplatelet Use	96 (48.0)
Duration between Pre-Op and Post-Op Measurements (Hours)	18.7 ± 10.5
Length of Surgery (Minutes)	80.5 ± 45.3
Pre-Op Hemoglobin/Hematocrit (g/dL)	12.6/37.4 ± 2.2/6.4
Pre-Op RBCs/WBCs	4.4/9.3 ± 2.7/4.2
Post-Op Hemoglobin/Hematocrit (g/dL)	11.6/34.6 ± 2.0/5.7
Hemoglobin < 7	2 (1.0)
Post-Op RBCs/WBCs	3.8/9.4 ± 0.7/4.0
Change in Hemoglobin/Hematocrit (g/dL)	−1.0/−2.8 ± 1.0/3.2
Treatment Type	
** DSA/No Treatment**	**62 (31.0)**
** Treatment**	**138 (69.0)**
Coiling	17 (8.5)
Coiling, Flow Diversion	3 (1.5)
AVM Embolization	5 (2.5)
Fistula Embolization	13 (6.5)
Flow Diversion	16 (8.0)
Flow Diversion, Fistula Embolization	1 (0.5)
Flow Diversion, Onyx Embolization	1 (0.5)
MVP Embolization	1 (0.5)
Onyx Embolization	17 (8.5)
Stenting/Angioplasty	16 (8.0)
Stenting/Angioplasty, WEB Embolization	3 (1.5)
Thrombectomy	27 (13.5)
Thrombectomy, Stenting/Angioplasty	3 (1.5)
WEB Embolization	15 (7.5)
Arterial Access	
Radial	67 (33.5)
Femoral	131 (65.5)
Sheath Size (French)	
5	60 (30.0)
6	51 (25.5)
7	7 (3.5)
8	71 (35.5)
Post-Op Disposition	
Floor	9 (4.5)
Home	11 (5.5)
ICU	170 (85.0)
Intermediate	8 (4.0)
Post-Op Complications	5 (2.5)
Death	1 (0.5)
Groin hematoma	1 (0.5)
ICH	3 (1.5)

**Table 2:** Ranges of hemoglobin decrease by treatment type, Pearson’s chi-squared test *p* < 0.0001.

Treatment Type	Hemoglobin Decrease (g/dL)		
	<1	1–2	>2
Diagnostic Angiograms (% of Total)	47 (75.8%)	11 (17.7%)	4 (6.5%)
Endovascular Interventions (% of Total)	56 (40.6%)	59 (42.8%)	23 (16.6%)

**Table 3:** Multivariate regression coefficients for hemoglobin difference. R^2^ = 0.25, *p*-value < 0.0001.

Variable	Estimate	Std Error	t Ratio	*p*-Value
Age (years)	−0.0099	0.0050	−1.97	0.050
Gender				
Female	−0.12	0.073	−1.64	0.10
Ethnicity				0.25 ^a^
Hispanic	−0.36	0.31	−1.16	0.25
Not Hispanic	−0.22	0.23	−0.94	0.35
BMI	0.0010	0.011	0.87	0.39
Pre-Operative Antiplatelet/Anticoagulant				
None	0.20	0.073	2.74	**0.0069**
Length of Surgery (minutes)	−0.0032	0.0018	−1.75	0.082
Treatment Type				
DSA/No Treatment	0.13	0.12	1.07	0.29
Femoral Access	0.033	0.091	0.36	0.72
Sheath Size				0.95 ^a^
5 French	−0.013	0.18	−0.07	0.94
6 French	0.044	0.15	0.29	0.78
7 French	0.052	0.29	0.18	0.86
Post-Op Disposition				0.11 ^a^
Floor	0.59	0.29	2.05	**0.042**
Home	−0.25	0.29	−0.87	0.39
ICU	−0.29	0.16	−1.79	0.075
Post Op Complications				
None	0.19	0.23	0.84	0.40

^a^ *p*-values for categorical variables with more than two levels were calculated from effect tests (Appendix A Table A1).

## Data Availability

Data available on request due to restrictions (e.g., privacy, legal or ethical reasons).

## Appendix A

**Table A1:** Effect Tests from Hemoglobin Difference Regression.

Variable	Sum of Squares	F Ratio	*p*-Value
Post Op Complications	0.6210223	0.7029	0.4030
Age at Encounter	3.4334950	3.8862	**0.0504**
Sex	2.3726727	2.6855	0.1032
BMI	0.6659901	0.7538	0.3865
Length of Surgery	2.7038987	3.0604	0.0821
Ethnicity	2.4589830	1.3916	0.2516
Treatment Type	1.0127829	1.1463	0.2859
Artery Access Site	0.1161092	0.1314	0.7174
Sheath Size	0.3251763	0.1227	0.9466
Antiplatelet/Anticoagulant Use	6.6142992	7.4863	**0.0069**
Post Op Disposition	5.4447867	2.0542	0.1083
